# Auto-LIA: The Automated Vision-Based Leaf Inclination Angle Measurement System Improves Monitoring of Plant Physiology

**DOI:** 10.34133/plantphenomics.0245

**Published:** 2024-09-11

**Authors:** Sijun Jiang, Xingcai Wu, Qi Wang, Zhixun Pei, Yuxiang Wang, Jian Jin, Ying Guo, RunJiang Song, Liansheng Zang, Yong-Jin Liu, Gefei Hao

**Affiliations:** ^1^ State Key Laboratory of Public Big Data, College of Computer Science and Technology, Guizhou University, Guiyang, China.; ^2^ Department of Computer Science and Technology, Tsinghua University, Beijing, China.; ^3^ Department of Agricultural and Biological Engineering, Purdue University, West Lafayette, IN, USA.; ^4^ School of Information Science, North China University of Technology, Beijing, China.; ^5^ National Key Laboratory of Green Pesticide, Guizhou University, Guiyang, China.

## Abstract

Plant sensors are commonly used in agricultural production, landscaping, and other fields to monitor plant growth and environmental parameters. As an important basic parameter in plant monitoring, leaf inclination angle (LIA) not only influences light absorption and pesticide loss but also contributes to genetic analysis and other plant phenotypic data collection. The measurements of LIA provide a basis for crop research as well as agricultural management, such as water loss, pesticide absorption, and illumination radiation. On the one hand, existing efficient solutions, represented by light detection and ranging (LiDAR), can provide the average leaf angle distribution of a plot. On the other hand, the labor-intensive schemes represented by hand measurements can show high accuracy. However, the existing methods suffer from low automation and weak leaf–plant correlation, limiting the application of individual plant leaf phenotypes. To improve the efficiency of LIA measurement and provide the correlation between leaf and plant, we design an image-phenotype-based noninvasive and efficient optical sensor measurement system, which combines multi-processes implemented via computer vision technologies and RGB images collected by physical sensing devices. Specifically, we utilize object detection to associate leaves with plants and adopt 3-dimensional reconstruction techniques to recover the spatial information of leaves in computational space. Then, we propose a spatial continuity-based segmentation algorithm combined with a graphical operation to implement the extraction of leaf key points. Finally, we seek the connection between the computational space and the actual physical space and put forward a method of leaf transformation to realize the localization and recovery of the LIA in physical space. Overall, our solution is characterized by noninvasiveness, full-process automation, and strong leaf–plant correlation, which enables efficient measurements at low cost. In this study, we validate Auto-LIA for practicality and compare the accuracy with the best solution that is acquired with an expensive and invasive LiDAR device. Our solution demonstrates its competitiveness and usability at a much lower equipment cost, with an accuracy of only 2. 5° less than that of the widely used LiDAR. As an intelligent processing system for plant sensor signals, Auto-LIA provides fully automated measurement of LIA, improving the monitoring of plant physiological information for plant protection. We make our code and data publicly available at http://autolia.samlab.cn.

## Introduction

**Fig. 1.**
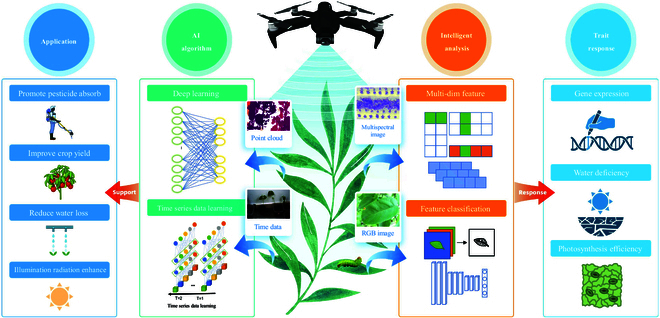
The application of AI algorithms helps expand the range of applications of LIA. As a self-regulated LIA, its physical traits can affect pesticide absorption, crop yield, water loss, and illumination radiation. LIA also serves as feedback on internal plant properties. Through intelligent analysis, it is able to assess the superiority of genes, monitor the water deficit status of leaves, and analyze the efficiency of photosynthesis.

Plant sensor, a kind of agricultural sensor that can monitor plant growth [1–9] and environmental parameter [10,11], is widely used in agricultural production [12–14], landscaping, and other fields. Plenty of plant phenotype information collected with plant sensors not only provides ample data for gene analysis [9,15–17] but also enables early detection [18,19] and intervention [20] for plant health issues. Lou et al. [15] point out that as one of the plant phenotypes, leaf morphology is an important factor affecting plant architecture, photosynthesis, and yields of cut chrysanthemums. Leaves, the vital organs of plants, feedback on the internal state of the plant and influence the environment [15,21] with their physical characteristics [18,19,22–24] as shown in Fig. 1. Leaf inclination angle (LIA), the angle between the leaf surface and the horizontal plane of the plant, is a crucial and fundamental parameter in plant phenotype data. For example, Zhang et al. [25] note that the LIA is mainly regulated by brassinosteroid and auxin signaling, which affects yield gain, reflecting the health status of plants. Moreover, as described in [26], accurate LIA measurements can help to calibrate hyperspectral images and improve the efficiency of imaging, suggesting that a proper solution can also improve the quality of high-throughput data measurements of other plant phenotypes. Therefore, the accurate LIA collection can facilitate the monitoring of plant growth status and also contribute to the regulation of the growth environment.

To achieve accurate LIA collection, there are some different methods to explore the LIA measurements. As shown in Table 1, existing LIA acquisition methods consist of traditional contact measurements and batch indirect measurements. The traditional contact measurements use an inclinometer [27,28] or a 3-dimensional (3D) digitizer [29], which is capable of precisely measuring the LIA parameters of every single leaf requiring tedious operations and stimulation of human contact. The other indirect measurements with the empirical equation [30–34], multidimensional environmental information [32], and radiation feedback [33–36] improve the efficiency of LIA measurement. The main reasons for the low level of automation in the above LIA measurement are that some collection methods are invasive [27], leaves can be hardly associated with plants, and leaf key points cannot be selected automatically. With the booming development of computer vision techniques [37], a rich variety of computer image-based methods are offered, which provide plenty of solutions for the functionality needed in LIA measurement [38]. However, these existing methods are still difficult to achieve a fully automated, noninvasive, and efficient measurement.

**Table 1.** Comparison of different LIA measurements in 3 dimensions: time-consuming, cost of equipment, and range of application. The time-consuming includes the time put into data acquisition and data processing. As for the application scenarios, we compare the precision of the measurements as well as the magnitude of the measurement objects. In comparison, our approach exhibits faster acquisition, lower cost expense, and stronger leaf correlation.

Type	Class	Time-consuming		Cost	Application	
		Acquire	Process		Granularity	Definition
Indirect	Commons [53]	Short	Short	High	Average	Plot
Direct	Hand-held [27]	Long	Long	Low	Single leaf	All biological
	LiDAR [22]	Short	Long	High	Average	Plot
	LDP [54]	Short	Long	Low	Average	All leaves in view
	Ours	Short	Short	Low	Single leaf	Individual plant

To this end, we propose an efficient LIA measurement system based on computer vision techniques and spatial geometric [39] transformation. It is a nondestructive, efficient, and more targeted acquisition system that improves the efficiency of LIA collection, reduces the manpower invested in the collection process, and avoids the harm of contact collection to plants. Motivated by the success of computer vision technologies, we consider a 4-stage multi-process solution to overcome the complex influences of LIA measurement. First, to solve the problem of invasive data collection, we choose RGB images as data input, which is characterized by a low-cost and noninvasive acquisition process. The support of RGB images for a variety of other agricultural applications, such as pest and disease detection and yield estimation, gives our system the potential to be integrated with other RGB image processing functions. Second, recovering the spatial information of the plant while preserving the leaf–plant association relationship inside the computational environment is the basis of LIA extraction. We utilize the object detection method to find the correlation relationship between leaves and plants, and apply the binocular reconstruction technique to implement the reconstruction of the spatial information of plants. Furthermore, as for the leaf key points, accurate segmentation of the image and morphological analysis of the leaves are key to automating the whole process. Finally, it is a challenge to estimate leaf-horizontal plane correlations in computational space. We add an inclinometer to capture the correlation between the imaging plane and the horizontal plane, and the LIA is captured through a coordinate transformation. These function components address the challenges to full process automation of LIA measurements from different perspectives, enabling low-spend, high-efficiency, and practical LIA acquisition.

Specifically, first, the hardware part of the system consists of 2 relatively stationary RGB cameras with an inclinometer, and a set of input data consists of 2 RGB images with the angle between the hardware and the horizontal plane. Then, we combine object detection, as YOLOv7 [40], and 3D reconstruction by RAFT [41] for recovering leaf phenotypic information in the imaging space, which is the basis for subsequent calculations. Next, as for the accurate extraction of the key points of leaves, we propose a surface segmentation based on the gradient of spatial depth and angles, which implements the precision segmentation for leaves. Finally, we put forward a method called leaf-image, which enables low-cost, batch-acquisition measurements of LIA through the correlation relationship between leaves, the imaging plane, and the physical space. We have collected a large amount of real data and have evaluated it with respect to the time consumption, accuracy, and practicality of the above schemes, which shows that Auto-LIA can efficiently address existing problems such as labor-intensive, intrusive acquisition, and low automation.

The advantages of our system lie in the following:

• To best our knowledge, we first propose an image-phenotype-based optical sensor measurement system for LIA, a low-cost and high-efficiency method with a strong relevance between leaf and plant.

• The noninvasive automation of the entire process is achieved through a multi-process approach, which dissects the structure of the leaves and reduces heavy labor.

• We propose a method of noncontact LIA measurement based on geometric transformations, which enables the batch output of angles and provides indicative ideas for measurements of leaf morphology.

• Our solution demonstrates its competitiveness and usability at a much lower equipment cost, with an accuracy of only 2. 5° less than that of the widely used light detection and ranging (LiDAR) schemes.

We expect that the introduction of Auto-LIA, a tool for fully automated LIA measurement, will improve the efficiency of LIA measurement and promote the development of intelligent agriculture. We also expect that it can provide a reference processing scheme for other plant phenotype data, which is difficult to achieve fully automated extraction.

## Materials and Methods

### Experimental Design

**Fig. 2.**
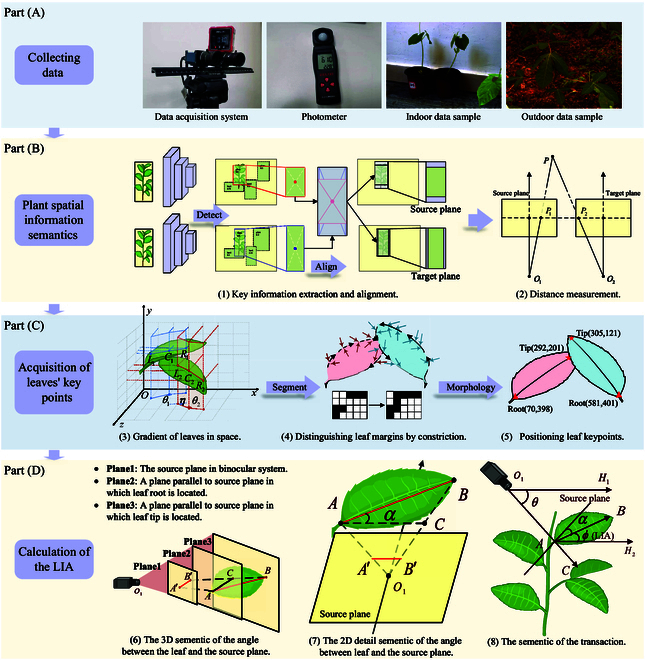
Pipeline of Auto-LIA’s data acquisition and processing. The system is composed of 4 main processes: data collecting, plant spatial information semantics, acquisition of leaves’ key points, and calculation of the LIA. (A) Hardware devices used in data collection. (B) Realization of the reconstruction of the plant in computational space. (C) Accurate extraction process of leaf key points. (D) Transformation relationship between computational space and physical space. The LIA is shown as *ϕ* in (8).

Indoor plants. Due to the importance of legumes and their widespread leaf similarity, research on LIA attributes of legumes is conducive to the advancement of multidimensional information collection in multiple fields of crops. In addition, the rich leaf morphology, high adaptability, and short growth cycle of legumes reduce the dependence of Auto-LIA validation on seasonal orders. It also enables us to obtain abundant phenotypic data with rapid validation in a short period of time. Thus, we select Tetraodonta seedlings grown from 7 d to 15 d, as shown in Fig. 2A, which cover the whole stage of bean seedlings from germination to pre-drafting. Specifically, we use the seed soaking and sprouting method to germinate young string bean seedlings, which lasts about 2 to 3 d. Then, sprouts are planted into pots, and after about 4 d, the seedlings grow cotyledons. Next, for approximately 7 to 9 d, plant seedlings develop rapidly, a stage at which we collect rich plant phenotypic data. Finally, at the time of their drafting, the plants are too tall to capture the entire plant with the same photographic parameters, so we stop collecting data.

Outdoor plants. To verify the practicability of Auto-LIA, we extend the experimental data-capturing environment and experimental subjects. As shown in Fig. 2A (Outdoor data sample), we choose castor grown in an outdoor environment as a sample, which is growing at Guizhou University (106.65°N, 26.45°E). We spend 2 d in an outdoor environment to collect and clean 50 sets of data with light intensity to measure the performance of Auto-LIA’s algorithmic accuracy. In detail, there is no wind at the moment of collection, the temperature of the environment is at 16°C, and the average ambient light is 5,416 lux. By comparing the performance of Auto-LIA in indoor and outdoor environments, its robustness and utility can be further quantified. In addition, windy environments may have a potentially negative impact on the usability of the collected data. Strong windy environments, when the plants are shaking violently, exceed the acquisition efficiency of the system’s image acquisition tool, which may lead to defocusing during data acquisition, resulting in a decrease in the usability of the acquired data.

Data acquisition system. The selection of the sensor determines the cost of the system and whether the LIA measurement is invasive. RGB camera, a noninvasive acquisition tool, which is widely used in life and with low cost, provides a good choice for LIA measurement. Meanwhile, RGB images provide data support for a large number of agricultural applications, such as disease identification [42–44], yield estimation [45], and so on. The choice of RGB cameras for data acquisition provides more scalability for the system. Since the use of RGB cameras for spatial reconstruction only captures the correlation between the source plane and the spatial points, it does not provide sufficient data support for LIA acquisition. We add an inclinometer to the acquisition system for measuring the angular relationship between the source plane and the level surface. This not only minimizes the invasiveness of manual measurements using inclinometers but also correlates the computational space with the ground. These 2 types of sensors form the source of information for the entire system.

Data collection. The focal length and wide-angle size of the camera limit the setting of the inclination between the system and the horizontal plane during LIA acquisition. If the shooting distance is fixed at 3 m, according to the imaging principle in reality, the plant can be fully photographed only when the angle between the LIA system and the horizontal plane is 18° to 38°. In order to verify the effect of the shooting angle on the measurements, we take values for the range uniformly over a span of 5°. Therefore, the data we collected are divided into 5 categories according to the shooting angle, which are 38. 7°, 32. 5°, 29.35°, 23. 4°, and 18. 6°. For the LIA measurement work, our data consist of 2 synchronized RGB images, the inclination from the data acquisition system to the horizontal plane, and the LIA of leaves under test. In the indoor environment, we employ 5 bean seedlings as materials and construct 5 data collection periods, which are separated by different shooting angles. In each period, we sample data on individual plants and collect data from 2 different viewpoints of a seedling by rotating the plants horizontally. In summary, for each plant, we take groups of images from 10 angles, and the indoor data collection process yields a total of 50 groups of data, i.e., 100 images, 50 shooting angles, and LIAs for all leaves can be recognized by the naked eye for accuracy assessment. As for the outdoor environment, 7 ramie plants make up our sample materials. We collect 10 pairs of data from different viewpoints, of which at least 3 plants are in each image. Moreover, the time of our data acquisition is limited to 0900 to 1700 every day so as to collect clear image information under natural light. Due to the time-consuming manual measurements of the LIA, and the quality of the captured images limiting the use of the data, it takes approximately 20 min to collect a valid piece of data after data cleaning.

Data processing. After the data acquisition system has been built, we fix the positional relationship between the 2 RGB cameras and calibrate them with the details described in the Supplementary Materials (Camera Calibration). The relationship is the basis for the subsequent depth estimation function. Next, during the data acquisition process, we capture the scene information synchronously with 2 RGB cameras in a relatively fixed position. An inclinometer is used for measuring the angle between the imaging plane and the horizontal surface, and manually collecting the actual LIAs in a very short time interval. Finally, all the data are aggregated together and computed with the evaluation platform.

Evaluation criteria. The evaluation metrics of this system focus on 3 aspects: processing time consumed, number of leaves recognized, and average error. The statistics of processing elapsed time mainly consist of the elapsed time from image input to batch LIA output, which is an indicator of the efficiency of data processing. The recognition rate of leaves is calculated by Eq. 1, which is a reflection of the effectiveness of the segmentation algorithm. *leaves_eva_* is the number of leaves, which are segmented, and *leaves_gt_* defines the leaves we can distinguish from the image:

$$\text{rate}=\frac{\text{leave}s_{\mathrm{eva}}}{\text{leave}s_{\mathrm{gt}}}\times 100\%$$ (1)

To verify the accuracy of the LIA acquisition, we adopt the metrics in Eq. 2 to estimate the accuracy of the acquisition process. *LIA_gt_* denotes the true value of LIA collected by the inclinometer, *LIA_m_* denotes the magnitude of leaf inclination estimated by Auto-LIA, and *dist* indicates the deviation of the measured value from the true value. In the statistical process, we use the difference between each recognized leaf and the true value as an evaluation metric of Auto-LIA.

$$\text{dist}=\left|\mathrm{LI}A_{\mathrm{gt}}-\mathrm{LI}A_{m}\right|$$ (2)

### Method

#### Overview

As shown in Fig. 2, the data acquirement system is displayed in part (A), and the framework of Auto-LIA is illustrated in part (B) to part (D). First, we use the binocular system described in the “Experimental design” section to capture images synchronously and measure the angle between the imaging plane and the ground using an inclinometer at the same time. Then, we reconstruct the spatial information in computing space with the scheme shown in part (B). Next, to extract the locations of key points of the leaves, we use both segmentation and morphological transformation of the images, as shown in part (C). Finally, part (D) shows the association between leaves, the imaging plane, and the horizontal surface, which is the theoretical support for Auto-LIA.

#### Plant spacial information semantics

In order to obtain complete information about the plant, we construe a spatial information perception method, which can provide refined data for LIA measurement. Specifically, the spatial information perception method consists of 2 parts: perception of semantic information of plants and reconstruction of spatial information. As shown in Fig. 2B (1), 2 techniques, object detection and image alignment, constitute the semantic perception of the plant. First, to obtain the semantic information, RGB images are fed into YOLOv7 [40] with its detailed parameters shown in the Supplementary Materials (Model Adaptation), following which the object type, coordinates, and confidence score in the images are output. Second, we iterate through all the detected objects and select the plant with the highest confidence so that the best quality plant in the image is included in the calculation. Third, we align the size by expanding in all 4 directions of the 2 selected boxes output above, details of which will be described in the Supplementary Materials (Plant Area Alignment). Fourth, we crop the target plants from the original images according to the position of the expanded target boxes. At last, the spatial information of the plant is reconstructed with RAFT [41] as shown in Fig. 2B (2), whose inputs are the cropped images of the source plane and the target plane and model parameters are detailed in the Supplementary Materials (Model Adaptation). On the one hand, the semantic perception of plants reduces the size of the images involved in the subsequent computation, which reduces not only the computation time but also the negative impact of ambient noise on the measurements. On the other hand, the reconstruction of spatial information achieves noninvasive spatial information perception and also reduces the equipment cost incurred.

#### Acquisition of leaves’ key points

In the whole process, the extraction of the key points of leaves plays an important role in the calculation of LIA, which is the basis of LIA calculation and the key to reducing the computational complexity and improving calculation efficiency. The extraction of leaves’ key points consists of 2 processes: the accurate segmentation of leaves and the acquisition of leaves’ keypoints, which are described in detail below.

Segmentation with the gradient of leaves in space. To provide accurate segmentation of leaves and reduce the negative effect of similar RGB messages on the segmentation, we propose a segmentation solution on the basis of the surface of leaves from the perspective of physical mechanics. Specifically, the leaves are quite thin and light with a tendency to bend, although the leaf surfaces of a single leaf will be within the same curved surface no matter how it is bent. Only when the force applied to the leaves exceeds the strength limit of the leaf, it will cause the leaf to fracture, resulting in 2 different curved surfaces. Thus, we adopt the angular transitions between neighboring pixel points in the depth map as the basis for surface resolution.

As shown in Fig. 2C (3), 2 leaves in the physical space are finely segmented depending on the gradient difference between neighboring pixels. Specifically, we take 6 representative points to describe the idea of our algorithm in detail. Point *L*_1_, point *C*_1_, point *R*_1_ and point *L*_1_, point *C*_1_, point *R*_1_ are labeled on leaf 1 and leaf 2, respectively. Generally, for all depth estimation, we utilize a Euclidean coordinate system to represent the spatial information of leaves. The *xOy* plane corresponds to the imaging plane, and the *z* axis represents the depth of the corresponding spatial point. Angle *θ*_1_ and angle *θ*_2_ respectively correspond to the projections of ∠*L*_1_*C*_1_*R*_1_ and ∠*L*_2_*C*_2_*R*_2_ on the plane *xOz*, i.e., the gradient values of the spatial depth. The magnitude of the gradient change of the spatial depth difference of neighboring pixels is adopted as a metric, with a global threshold *segTh* set to implement the segmentation of leaves, and for enhancing the robustness of the algorithm, we also define a global anti-noise threshold *nTh* called jump pixels. When the gradient is greater than the specified threshold *segTh*, the 2 points are considered to be in the same plane, as *θ*_1_ and *θ*_2_. Conversely, the 2 points are considered to be in different surfaces, as angle *η*. Overall, according to the above principle, we achieve accurate segmentation of leaves. Compared with threshold-based segmentation and deep learning-based segmentation algorithms, our method realizes single-leaf-based segmentation, which provides a computational basis for the next step of accurately measuring the angle of each leaf.

**Fig. 3.**
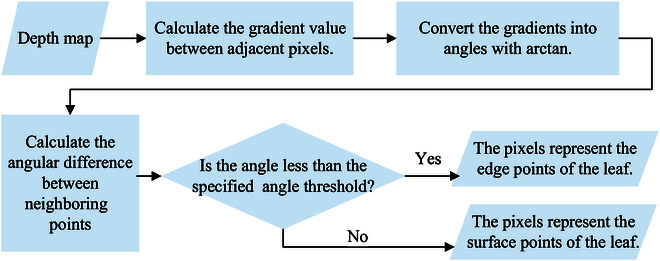
Schematic diagram of gradient-based segmentation. First, the gradient difference between neighboring pixel points in the depth map is extracted. Next, the gradient values are converted into angle values with respect to the imaging plane. Finally, the correlation between the angle difference between neighboring pixel points and the prescribed threshold is compared. This correlation relationship is used to determine whether each pixel point is a leaf edge point or a leaf surface point.

From the perspective of implementation, the process of leaf segmentation is shown in Fig. 3. After the 4-fold transformation of depth map–spatial gradient map–angular map–angular difference map, the pixels in the depth map are divided into 2 categories: leaf edge points and leaf surface points. We parse for the segmentation map, and the leaf surface points of the same leaf are clustered in one area. We use consecutive leaf edge points as the criterion for segmentation, and each region surrounded by leaf edge points is regarded as a separate leaf. Eventually, as illustrated in Fig. 2C (4), to accurately distinguish the region to which a leaf belongs, we use the region size as a criterion to filter other regions.

Extraction of leaves’ key points with graphical morphology. In order to reduce the complexity of the calculation and improve the accuracy of the LIA measurements, we locate the 2 key points involved in our process. According to the LIA definition, there are 2 key points: the leaf tip point and the leaf root point, as shown in Fig. 2C (5). We process each leaf region with the morphological open operation to obtain the venation of a leaf, which implies the leaf tip and leaf root. The open operation can be described as follows: *A* ∘ *B* = (*A* ⊖ *B*) ⊕ *B*, where the symbols ∘, ⊖, and ⊕ indicate the open operation, the erosion operation, and the dilation operation in computer graphics, respectively. For the purpose of simplifying the calculation, we specify that the point where the leaf vein is closest to the top of the image is the leaf root point and the bottom point is the leaf tip point. Combining the depth information of these 2 key points of a leaf to implement the LIA measurement will be described in the next section.

#### Calculation of the LIA

In order to automate the whole process of noninvasive LIA measurement, it is indispensable to transform the measured parameters from the plant in the reconstructed space to the real world. In this system, leaves are indirectly associated with the horizontal surface through the imaging plane, so the description in this section will be organized around 2 aspects below: the angle calculation in camera coordinate and the angle conversion between the imaging plane and the horizontal.

Angle calculation in camera coordinate. The imaging plane is a bridge between the captured image and the real scene. To construct a complete automated LIA measurement process, we quantify the basis for the interconversion of leaf physical and imaging information. LIA, a real physical property, is calculated after an intermediate parameter: the angle between the leaf and the imaging plane. The correlation between the imaging plane and a single leaf is shown in Fig. 2D (6). Point *A* and point *B* represent the leaf root and leaf tip, respectively. *O*_1_ is the optical center of the camera’s reference plane. In Fig. 2D (7), after connecting line *O*_1_*A* and line *O*_1_*B*, the source plane and 2 lines intersect at points *A*′,*B*′, which are the projection points of points *A*,*B* on the imaging plane. Line *AC* is parallel to line *A* ′ *B*′ through the point *A*, and the angle *α* is the angle between the leaf and the imaging plane that we want to obtain. Then, according to the law of cosines, *α* can be converted by Eq. 3:

$${\left|\vec{\mathrm{AC}}\right|}^{2}+{\left|\vec{\mathrm{AB}}\right|}^{2}-{\left|\vec{\mathrm{BC}}\right|}^{2}=2{\left|\vec{\mathrm{AC}}\right|}^{2}\cdot \left|\vec{\mathrm{AB}}\right|\cos\alpha$$ (3)

In addition, *d_A_* and *d_B_* denote the distance from point *A* and *B* to the imaging plane.

Then, *AC* can be calculated as follows: $\left|\vec{\mathrm{AC}}\right|=d_{A}\cdot \left|\vec{A\prime B\prime }\right|$. As for *BC*, Eq. 4 and the relationship $\vec{\mathrm{BC}}=\vec{O_{1}C}-\vec{O_{1}B}$ simplifies the calculation.

$$\frac{\left|\vec{O_{1}C}\right|}{d_{A}}=\frac{\left|\vec{O_{1}B}\right|}{d_{B}}$$ (4)

Another key parameter, $\left|\vec{\mathrm{AB}}\right|$, can be computed with Eq. 5, where ∠*AO*_1_*C* is the angle made by the center of light with the projection plane:

$${\left|\vec{\mathrm{AB}}\right|}^{2}={\left|\vec{O_{1}C}\right|}^{2}+{\left|\vec{{\mathrm{AO}}_{1}}\right|}^{2}-2\left|\vec{O_{1}C}\right|\cdot \left|\vec{{\mathrm{AO}}_{1}}\right|\cos∠AO_{1}C$$ (5)

The above equation is the theoretical support for the angle between the leaf and the plane. Combining the position of the leaf key point on the projection plane with the 3D data of the leaf key point in the virtual space, we are able to obtain the specific value of the key angle *α*, which is a crucial input for the next stage.

Angle conversion between imaging plane and horizontal surface. In order to implement the LIA measurement, we need to incorporate the intermediate parameters mentioned above with the inclinometer readings and convert them to get the measured values. In Auto-LIA, we replace intensive labor inputs with spatial geometric transformations. As shown in Fig. 2D (8), *BH*_2_ and *O*_1_*H*_1_ are 2 lines parallel to the ground plane, respectively, which is a strong correlation between the binocular system and the real scene in which the plant is located. In detail, *O*_1_ is the optical center of the source camera, while points *A*, *B* define the leaf root and tip of the target leaf, respectively. The link between the virtual and the real scene is the parallelism with the horizontal plane of line *O*_1_*H* and line *AH*. LIA *ϕ*, defined as the angle between the leaf surface and the ground plane, is determined by the angle between *AB* and *AH*_2_.

As shown in Fig. 2D (8), to visualize the geometric transformation between the binocular system and the real scene more intuitively, we apply the straight line *l* to represent the source plane. *O*_1_*C* is the line from the optical center to the center of the imaging, perpendicular to the imaging plane. With the theorem that 2 straight lines are parallel and same-side interior angles are complementary, LIA can be calculated as follows:

∠θ+∠α+∠ϕ+90°=180° (6)

So far, we have realized the transition from the intermediate perspective to the target LIA and have achieved the noninvasive acquisition of LIA.

## Results

### Visualization

**Fig. 4.**
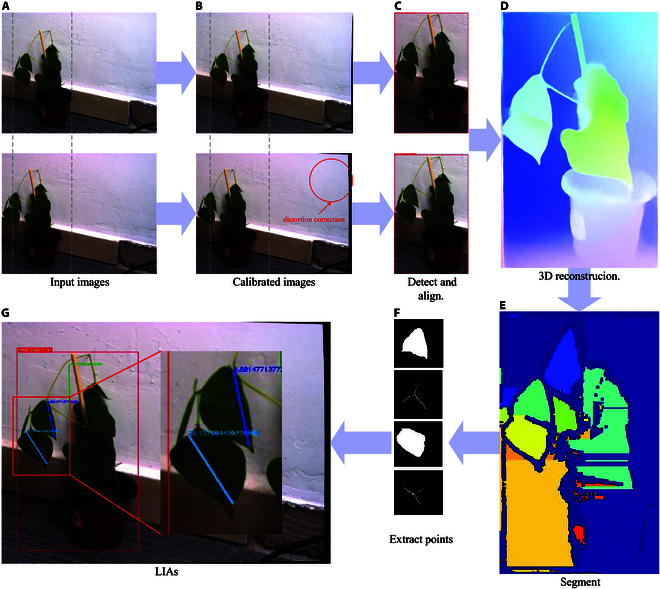
Process visualization of Auto-LIA. (A) Two synchronous images of the input system. (B) Two calibrated input images. (C) Two cut images without losing too much semantic information. (D) Depth image of reconstructed (C). (E) Segmented area of the scene. (F) Schematic diagram of leaf vein extraction with leaves’ regions. (G) Result of Auto-LIA’s measurement. The whole visualization proves the soundness of our approach.

The visualization of the process facilitates the analysis of the availability and effectiveness of each process and the timely adjustment of the implementation of the program. Therefore, we record the intermediate process of Auto-LIA’s data processing flow and the experiment shows that our operation is explainable. Figure 4A shows 2 synchronous images of the input system. Due to the relative distance between the 2 cameras, the same plant is in different positions in the images. Figure 4B illustrates 2 calibrated input images. After calibration, the image is deformed compared to the original. Figure 4C shows that the left and right images have been cut after detection and alignment without losing too much semantic information after the alignment operation. In Fig. 4D, the depth image obtained by converting the disparity map detailed in the Supplementary Materials (Transform of Disparity Map), whose shape is similar to the target plant stored in the computational space, is shown. Figure 4E shows different regions of the image cut out according to the depth information, in which the leaves that can be distinguished by the naked eye are divided into different regions. Since the segmentation method proposed in this paper is more sensitive to the depth falloff at the edges of the object, the overlapping leaves and the continuous untextured background region are more likely to be partitioned into the same region as shown in Fig. 4E. To minimize the effect of extra regions, we add a filtering operation as post-processing after segmentation so that Auto-LIA can localize leaf regions more precisely.

Figure 4F is a schematic diagram of leaf vein extraction. Finally, we draw the LIA according to the position of the veins in Fig. 4G, and the LIA corresponding to the 2 veins is output. After all, each of the above steps has fully realized its target function, thus making our system more perfect.

### Functional verification

**Fig. 5.**
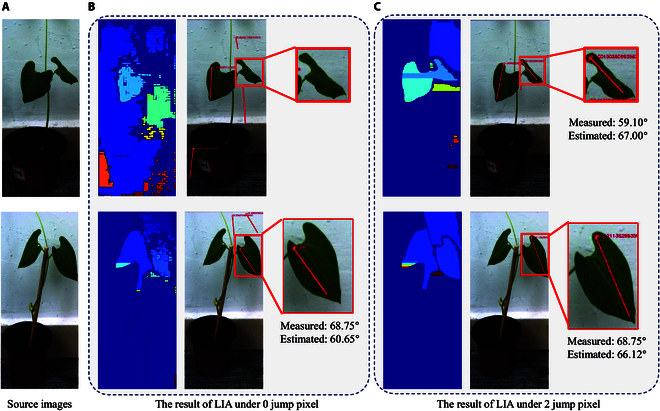
Visualization of functional effects. (A) Two cropped source images are used for LIA estimations. (B) The segmentation results and LIA measurements are obtained by running the program without denoising operations. (C) The leaf results are obtained by running the program with the jump pixels set to 2. The set of images demonstrates that the denoising operation has a positive effect on leaf segmentation as well as on LIA measurements.

To verify the role played by different functions within the Auto-LIA system, we disable the detection, jump pixel 2 auxiliary functions, and record the effect under different conditions. As shown in Table 2 and Fig. 5, the complete combination of these 2 methods demonstrates differential effects in terms of measurement accuracy and computation time. In detail, for the detection operation, it provides plant–leaf association and has a positive effect on the recognition rate, average error, as well as computation time, as demonstrated in Table 2. The reason is that the detection operation optimizes the measurement process of Auto-LIA from 3 perspectives: retaining semantic information, decreasing noise interference, and reducing computation. First, an anchor frame retains all the leaves on the same plant in a single image, preserving the association between the leaves and the plant. The algorithm used for stereo matching remains unchanged in this process, so both time and space complexity remain unchanged, but the size of the inputs involved in the computation is reduced. Second, the detection operation reduces the influence of irrelevant background on the spatial reconstruction, i.e., unimportant occlusions, thin structures, and other information that would substitute for inaccurate references are reduced. Finally, the pixels outside the anchor are no longer involved in the subsequent computation by cropping, effectively improving the computational efficiency and reducing the data processing burden.

**Table 2.** Semantic of LIA measurement when using different functions. We verify the influence of the detection method and jump pixel in the measurement process. The evaluation statistics of the results include recognition rate, average error, and calculation time, which are marked as RR, AE, and CT respectively.

Number of leaves	Detected	Jump pixel	RR	AE	CT
Multiple	True	2	100.00%	12.06	3.69
		0	50.00%	20.65	70.65
	False	2	50.00%	39.74	11.58
		0	50.00%	25.10	305.00
Single	True	2	100.00%	2.63	3.49
		0	100.00%	8.1	75.39
	False	2	0.00%	-	9.60
		0	100.00%	8.1	322.00

In addition, as far as the denoising operation jump pixel is concerned, its application facilitates the improvement of computational efficiency and measurement accuracy. As shown in Fig. 5, the leaf without using jump pixel is divided into several discrete small regions by the noise. On the one hand, a large number of discrete small regions may cause a large amount of memory occupation during leaf region comprehension, which leads to a linear increase in computation time. On the other hand, these small regions demonstrate inaccurate segmentation of the leaf region, which could cause inaccurate extraction of leaf key points and ultimately inaccurate LIA measurements.

### Recommended parameters

**Fig. 6.**
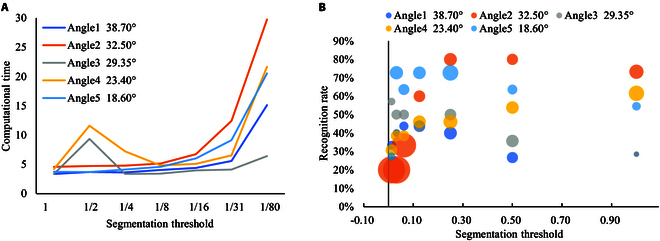
Auto-LIA performance under different segmentation thresholds and different shooting angles. (A) Line plots of segmentation thresholds versus computational elapsed time. (B) Bubble plots between segmentation threshold and leaf recognition rate, and mean deviation of measurements.

To verify the influence of different segmentation thresholds and shooting angles on the Auto-LIA system, we select the segmentation threshold in a multiple relationship, decreasing from 1 to 1/80. The effect of shooting angle and segmentation threshold on LIA acquisition is shown in Fig. 6. Under different segmentation thresholds, the recognition rate, average error, and average computational time of each set of data are counted.

On the one hand, the computation elapsed time shows an overall upward trend as the segmentation threshold decreases, while the elapsed time between different shooting angles shows a similar effect. This indicates that the computation time has some correlation with the setting of the segmentation threshold, while the correlation with the shooting angle is weak. The leaf vein extraction implements an open operation for each leaf region, and the time complexity of this operation reaches *O(n)*, which is positively related to the number of leaf regions involved in the computation. At smaller thresholds, the more subtle gradient changes of the leaf will be more easily captured, resulting in an excessive number of segmented regions during segmentation. Excessive segmentation regions lead to a rise in the number of leaves involved in the computation, which reduces the computational efficiency of the system.

On the other hand, the recognition rate of leaves is closely associated with the shooting angle and threshold of the system. The trend of the data demonstrates that the shooting angle has a greater effect on the recognition rate, while the segmentation threshold is more effective on the accuracy. The shooting angle affects the complete reconstruction of leaf spatial information, and the segmentation threshold affects the accurate localization of leaf key points. Therefore, we take 32.5^°^ as the shooting angle with 0.5 as the segmentation threshold while adapting the Auto-LIA system to the real scene.

### Accuracy

To assess the accuracy of LIA measurements, we conduct experiments in an indoor environment. Specifically, we gather data indoors using a data acquisition device and simultaneously measure the true LIA of the leaves using an inclinometer, which constitutes a piece of data. The collected data are cleaned and classified into 5 groups according to the shooting angle, and each group consists of 10 pieces of data. In the data processing stage, we feed each piece of data into the system in turn. After that, we record the average error of each piece of data under different segmentation thresholds, pixel filter sizes, and jump pixels with Eq. 2. Finally, we count the minimum error of each piece of collected data under its optimal parameters and calculate the average error of each group.

As shown in Table 3, the smallest average error is 5.42°, obtained at a shooting angle of 32.50°, while the current optimal LiDAR-based scheme reaches 2.5° [46], which indicates the competitiveness of Auto-LIA. Meanwhile, these results demonstrate a strong correlation between the measurement accuracy of the system and the shooting angle, and we believe that this may be attributed to the following 2 reasons. On the one hand, with the adjustment of the lens, the light reflection of the background affects the information collection of the scene. In the process of data cleaning, we find that there are spots in some RGB images. These images are performed poorly in the process of 3D reconstruction, and there are a lot of mismatches, which lead to inaccurate depth estimation and directly affect LIA acquisition. On the other hand, the angle between the imaging plane and the leaves might lead to a different occlusion of the leaf, which has a serious impact on the RGB information collected by the lens. The ill-posed problem seriously affects the accuracy of the depth estimation of the leaf, which is currently a hot issue for continuous optimization in 3D reconstruction. The problem of occlusion caused by the shooting angle is also widespread in other LIA measurements such as LiDAR and LDP. Overall, our method provides acceptable accuracy, and with proper adjustment, our method can be applied to real-world scenarios.

**Table 3.** Evaluation of the accuracy of LIA measurements. The experimental data are divided into 5 groups according to the angle between the imaging plane and the horizontal plane. The measurement accuracy of the system is optimal when the average error reaches the minimum, and its optimal measurement accuracy is obtained in group 2.

Group	Shooting angle	Measured average	Estimated average	Average error
No.	*θ*	*LIA_gt_*	*LIA_m_*	*dist*
1	38.70	61.17	63.25	6.90
2	32.50	59.70	63.83	5.42
3	29.35	57.06	61.77	8.07
4	23.40	52.56	50.57	10.25
5	18.60	62.16	75.77	13.61

### Practicality

In order to verify the practical effectiveness of Auto-LIA, we carry out validation and testing indoors and outdoors. Based on noninvasive considerations, the shooting distance needs to be more than 2 m away from the plant being photographed. To capture the complete plant while maintaining the shooting distance, the shooting angle is limited to 25.6°. We compare the luminosity and optimal accuracy of the outdoor and indoor scenes at similar shooting angles to verify the effect of different environments on Auto-LIA measurements.

**Fig. 7.**
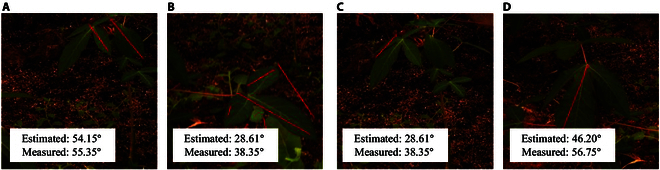
(A to D) Performance in practicality. We show the results of LIA measurements for different plants in the same environment. With ambient light, the photos captured by the camera are warm in tone. The examples show that Auto-LIA is able to properly measure in outdoor environments as well.

The LIA measurement results for the data of the outdoor environment are presented in Fig. 7, while the LIA estimation results for the data of the indoor environment can be referred to Fig. 5. On the one hand, as shown in Fig. 7, the measured results in (A) show a small difference from the estimated results, while the difference is larger in (D). The difference is mainly caused by the hardware equipment, nonideal correction, and viewpoint occlusion during the 3D reconstruction process, which affects the accuracy of the depth estimation. The serialized process design leads to the result of depth estimation directly affecting all subsequent calculations. This is an unavoidable and continuously optimized problem that needs to be addressed constantly as deep estimation evolves. On the other hand, apart from the difference, the hue and depth information in various environments present different effects, but the LIAs of the leaves are all able to be figured out successfully. The aforementioned statement suggests that the influence of diverse data acquisition environments on LIA estimation is minimized, thereby enhancing the robustness of our system.

We categorize the evaluation subjects into 3 groups as shown in Table 4, with the basis of the shooting angle and the environment.

**Table 4.** Results of the real and estimated LIA at different shooting angles. The environment around the plant includes the scene around the plant and the light of the environment. The average error of LIA measurements varies less in dissimilar environments. As a practical validation group, LIA measurements in outdoor environments are shown in bold.

Group	Environment			Shooting angle	Average error
	Surroundings	Light intensity	Temperature		
1	Indoors	315	18∘C	23.40	10.25
**2**	**Outdoors**	**5,416**	**16**∘**C**	**25.60**	**10.21**
3	Indoors	280	18∘C	29.35	8.07

There is a large gap in environmental parameters between group 1 and group 2, i.e., the light intensity in group 2 is 17.19 times higher than that in group 1, and the temperature difference is 2°C, but the average error is 99.6% similar to that in group 1 with a similar shooting angle. This indicates the robustness of the Auto-LIA system itself for data acquisition in different environments. Meanwhile, the data in groups 1 and 3 are taken in similar environments, where light intensity is close to 300 lux and temperatures are both close to 18°C at different shooting angles, but there is a 2.18° bias in their measurement accuracy. The data suggest that the source of the larger average error is introduced primarily by the shooting angle in the case where only the shooting angle differs.

The 2 sets of controls above demonstrate that the shooting environment is not the main factor affecting the Auto-LIA measurement accuracy, but the shooting angle could be the major factor.

### Monitoring of water deprivation status

**Fig. 8.**
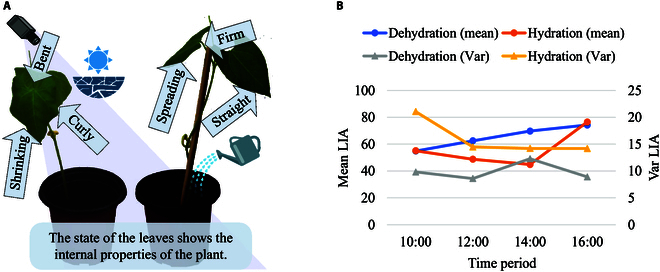
Association of LIA with plant physiological status, taking hydration as an example. (A) The plant on the left demonstrates the characteristic appearance of bent, shrinking, curly leaves under conditions of water deprivation. The plant on the right shows how the leaves look when well watered. (B) Changes over 8 h in the different plants’ LIAs. The mean LIAs of the 2 groups show different tendencies of change with time. The variances of LIAs for each group present the distribution of LIA in different time periods.

In order to validate whether LIA can provide feedback on the physiological state of the plant [47], we take the water deprivation status of the plant as an example. In detail, building on the experimental process in the “Experimental design” section, we control the water intake of the plants and metric the mean LIA with the variance (Var) of the LIA at each time period. First, we choose 4 pots of bean seedlings and equally divide them into 2 groups: the dehydration group and the hydration group. For the hydration group, we water them every day at 9:00 AM to ensure enough water intake for the plants. As for another group, we keep them from watering for 4 d to reduce their hydration content. Next, after 4 d, the states of the plants of the dehydration group and hydration group are shown in Fig. 8A. Finally, we count the mean LIA and the variance of LIAs between 9:00 AM and 5:00 PM and organize the data in 2-hour intervals. As shown in Fig. 8B, the mean LIAs produce a more drastic change over time in the well-watered condition, whereas the change in the water-deprived group is more sluggish.

## Discussion

The phenotypic information of leaves is closely related to the inner state of the plant. The presence of water deficiency in the plant [47], the presence of diseases in the plant [4], and the state of vitamin deficiency in the plant can be expressed by the morphology of the plant leaves. The LIA is an important quantitative basis for population classification [21], genotypic superiority and inferiority analysis [15], input loss estimation [48], and growth status monitoring [47] of plants. However, current widely used LiDAR solutions [22] require high equipment costs and high collection granularity, while hand-holding measurement [27] is labor-intensive. Auto-LIA provides a low-cost and short time-consuming solution for LIA collection that can correlate leaf and plant information, which is essential for precision agricultural development.

### Biological relevance

As shown in Fig. 8, LIA in various physiological states within similar growth stages shows different trends over time. The retarded changes can be attributed to the fact that the synthesis and distribution of plant hormones are affected under drought conditions, which in turn affects leaf growth and morphological changes. At the same time, the variances of LIAs for each time period in the well-watered subgroup are always larger than those in the drought subgroup. These trends indicate that the distribution of LIA in the well-watered subgroup is more discrete, and the plants are more sensitive to externally generated stimuli. Overall, the measurement of LIA by Auto-LIA can reflect plant physiology and further improve the accuracy of monitoring plant health.

**Fig. 9.**
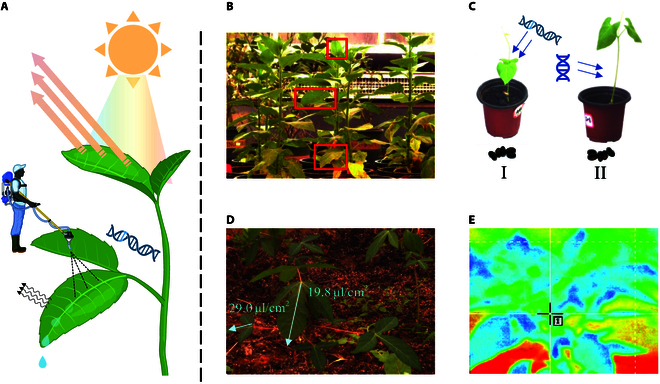
Expansion of LIA applications. (A) LIA influences light absorption and pesticide loss, and also contributes to genetic analysis and other plant phenotypic data collection. (B) LIA influences light absorption and reflection. (C) LIA is one of the reference values for crop genetic superiority analysis. (D) LIA influences the amount of loss during pesticide spraying. (E) LIA affects the accuracy of data collection for other plant phenotypes.

In addition, Auto-LIA, a low-cost, noninvasive LIA collection solution, extends agricultural applications. As shown in Fig. 9A, LIA is one of the bases for analyzing plant genetic traits nowadays, while part of the application relies on LIA to have an effect.

First, as shown in Fig. 9B, LIAs at various heights of the same plant have different absorption and reflection efficiencies of light energy, which can be applied to precision agricultural management to improve crop yields. In the traditional way, the precise collection program on a single plant basis requires a large human input and is time-consuming, and human activities may cause irritation to the plant. On modern farms, timely data collection and effective environmental feedback serve on crop yields’ enhancement. The low-cost, noninvasive, strong-correlation Auto-LIA system achieves an environmentally sustainable collection of LIA, which improves continuity and refinement of agricultural management. Therefore, we believe that with the gradual promotion of Auto-LIA, mechanisms for precise agricultural management, such as LIA-based controlling of light angles, would be established to enhance crop yields.

Second, some researchers believe that LIA is a manifestation of crop genetic superiority [15], which relates to organic production. Figure 9C shows the application of LIA in breeding analysis nowadays, which is based on the theory that superior gene expression synthesizes more organic matter by regulating LIA. Auto-LIA supports the noninvasive collection of data while continuously and automatically obtaining the data, and the original frequency of the collection of LIA can be improved. For complex genetic information analysis, more massive data and more intensive collection frequency are conducive to improving the accuracy of plant genetic analysis. The deployment of a large number of Auto-LIA systems enables richer plant growth data to be fed back, which provides multidimensional and refined information reference for the intelligent breeding process.

Third, as shown in Fig. 9D, with plant protection becoming a hot topic in recent years, the negative correlation between LIA and pesticide loss rate [49] has gradually come into researchers’ sights. Auto-LIA, a highly efficient LIA collection method, can quickly measure the LIA value of the target plant. The use of refined spraying for different LIA is conducive to improving the utilisation rate of pesticides, which also leads to a reduction in the negative impact of agricultural inputs on the environment. We expect Auto-LIA to become one of the foundations of pesticide precision spraying technology, providing technical support for efficient pesticide spraying.

Finally, as described in [26], the accurate measurement of LIA has a facilitating effect on the acquisition of other plant phenotypic information. In addition to the visible light region, the invisible light field represented by Fig. 9D also provides abundant data for the precise measurement of plants. We believe that the proposal of Auto-LIA not only is a means of data acquisition but also provides a facilitating role for the measurement of other data.

Furthermore, we hope that as the difficulty of LIA collection decreases, LIA will become the basis for more research and more agricultural applications will emerge.

### Equipment selection

The noninvasive and low-cost acquisition solution provided by Auto-LIA offers the feasibility of fine-grained continuous monitoring of LIA. Compared with the LiDAR solution, which constantly emits laser particles into the environment, the RGB camera solution is less invasive, has lower equipment cost, and is more scalable. On the one hand, the equipment cost of the widely used RGB camera is much lower than that of LiDAR, which controls the cost investment of monitoring to a considerable extent. On the other hand, RGB images play an important role in agricultural applications. Using images as an information source can expand the functions of data processing systems, such as disease detection and yield prediction, without increasing acquisition equipment. Nevertheless, the adoption of RGB images as a data source also brings some problems, such as the information collected is easily affected by ambient lighting. The accuracy of indoor plant and outdoor environment measurements is shown in Fig. 7 and Table 4, and Auto-LIA shows less fluctuation in measurement accuracy under different light intensities. As these data shown, the scheme Auto-LIA suffers from some light robustness in ensuring that the plant leaves are clearly visible. Due to the characteristics of RGB imaging, we still need to avoid unclear imaging during acquisition. Unclear imaging will result in a marked loss of information carried in the image, leading to a decrease in estimation accuracy.

### Technical analysis

As shown in Fig. 6, the different technologies used in Auto-LIA enhance the measurement in terms of both functionality and performance. In particular, the detection function enables the localization of the target plant and preserves the association between the plant and the leaf, which enables targeted monitoring of LIA for a single plant. Nonetheless, serialized processes, which link the detection function to subsequent subprocesses, increase the dependence on the plant being successfully targeted. The accuracy of the detection will have a direct impact on the measurement of the LIA value of a single plant. In dense plant environments, when multiple plants are retained in the same detection frame, it also leads to some misleading information about the collection of LIA for a single plant.

The “jump pixel” operation reduces the noise transfer in the overall data processing flow of the Auto-LIA system to a certain extent. However, denoising operations can also lead to loss of detail during image processing, due to the thinness of the leaves perpendicular to the lens, which can be easily mistaken for noise and ignored during segmentation. With technological innovation, existing problems will be gradually optimized or even solved, and the LIA measurement will be further optimized to meet the needs of precise agricultural management and discipline data collection.

Seen from the perspective of the entire computational process, the operational approach to data processing is completely linear. There is a serial relationship between the 3 processes of detection, depth estimation, and segmentation so that the overall computational time consumption is the sum of these 3 processes. In terms of individual algorithms, YOLOv7 and RAFT are both convolutional neural network (CNN)-based deep learning algorithms that have smaller model sizes and are faster to compute, but use more memory and CPU cores. In terms of individual algorithms, both YOLOv7 and RAFT are CNN-based deep learning algorithms, which have smaller model sizes, faster computation speeds, and frameworks that provide a high degree of parallelization, which use more memory resources. In this study, a machine with only 32C and 40G of RAM is all that is needed, and a server of the same configuration charges an economical $0.75 per hour. In this study, it can be implemented by a machine with just 32C and 40G RAM, which corresponds to the same configuration of the server, and its computational price is 5.43 per hour, which is very economical. The “acquisition of leaves’ key points” proposed in this study is the algorithm that most affects the computational speed in the current computing process. Among them, the adjacent region fusion and labeling method requires 2 traversals of the depth map. Although the computational complexity of this traversal operation is *O(n)*, its computational time is linearly related to the number of image pixels involved in the computation and is logically difficult to parallelize, which leads to a longer computational time. However, there is no conflict between the resources used by these 2 types of operations, and there is less conflict between the high-speed, high-resource deep learning algorithm and the low-speed, low-occupancy segmentation algorithm with respect to the utilization of resources under parallel conditions. By means of parallel invocation, the utilization of computational resources and the average response rate of computation can be effectively improved.

### Error analysis

As an image-phenotype-based noninvasive and efficient optical sensor measurement system, Auto-LIA combines multi-processes implemented via computer vision technologies and RGB images. For the whole system, the error is mainly introduced by 3 processes: detection and localization, depth estimation, and gradient-based segmentation.

Key information extraction and alignment. In this study, we use a confidence score of 0.6 to filter regions, i.e., when the confidence score is less than 0.6, the corresponding alternative region is filtered. In addition, of all alternative regions above the set confidence threshold, the one with the highest score will be selected. Designing an accurate threshold to achieve the filtering of the target may result in some plants being missed, which leads to a reduction in the final recognition rate. From the perspective of design, the exact threshold to achieve target filtering may result in some plants being missed, which leads to a reduction in the final recognition rate. However, during the construction of the dataset provided in this study, we find that the better-quality plants have confidence scores centered above 0.8. The images with confidence levels lower than 0.6 generally suffer from overexposure and artifacts, as well as the problem that the detected image with the highest confidence level between the left and right images does not belong to the same plant, which is very unfavorable for the use of subsequent distance measurement submethods based on RGB images.

Distance measurement. Building on the noninvasive character of stereo matching to measure spatial depth, we combine disparity mapping with a binocular system to accomplish depth measurements. The RAFT algorithm used in this paper is lightweight and can be easily deployed on mobile devices. However, due to the difficulty in acquiring stereo matching datasets, our evaluation of the model properties can only refer to the performance on the largest stereo matching dataset available for real-world scenarios: the KITTI-2015 [50]. On this dataset, the RAFT algorithm is able to achieve a percentage of erroneous (EPE > 3.0 px) foreground pixels among published methods, i.e., the number of pixels with an estimation bias of more than 3 px during the estimation process averages at 2.96%. It is clear that this dataset has a large and unquantifiable domain gap with the data in the agricultural scenario, which has a profound and unmeasurable impact on our measurements. The depth estimation session is arguably the most influential part of the whole process, as incorrect disparity estimation may cause the accuracy of the results of the later serial spatial-based segmentation methods. In order to minimize the impact of disparity estimation on the whole process, we add a preprocessing process to the distance metric, which assists in reducing the impact of continuous mismatch points in complex scenes on the wrong matching of key plant regions by means of detection. Meanwhile, in the subsequent processing, we use a unique segmentation scheme to reduce the impact of discrete mutant mismatch points on LIA measurements.

Acquisition of leaves’ key points. The segmentation based on gradient provides an efficient tool for location leaves.

The advantage of this algorithm, where the segmentation process is serialized with the depth estimation, is that the edges are always on the same surface. For final measurement accuracy, Auto-LIA implements segmentation based on the angular difference between neighboring points in a straight line, for which a threshold value can be set. When an instantaneous increase in the rate of change of the angle between neighboring pixels occurs and is greater than the set angular threshold, the portion is recognized as being on another surface, thereby reducing the effect of abrupt changes in edges and segmentation errors on the overall abnormally high value of the measurement. The adverse effect of the accuracy of the segmentation operation on the final result of the LIA measurement is limited to a fixed range. As a result, the error in the final measurement arises more from inaccuracies in the distance measurement than from the accuracy of the segmentation itself.

### Application scene and challenge

Auto-LIA, as a plant phenotypic information collection system, can be cost-effectively integrated into larger monitoring platforms and combined with other collection systems to realize multi-faceted monitoring of plant physiological status and promote more precise agricultural management. In terms of pesticide application, Lu et al. [49] investigated the correlation between pesticide application loss and LIA. The study shows that the spraying angle of pesticides can have an effect on the amount of pesticides absorbed by plants.

As one of the upstream tasks of pesticide precision application, Auto-LIA provides a fine-grained plant phenotype data collection solution that can provide data reference for precision spraying. Combined with mechanized and automated spraying equipment, Auto-LIA is capable of assisting in reducing the waste of agricultural inputs. From the perspective of agricultural management, Auto-LIA provides results for agriculture in terms of both environmental protection and cost reduction.

Nevertheless, as an emerging technology, Auto-LIA still has a lot of improvements to be made. For example, during the collection process, the problem that the target plants cannot be detected as a result of the large domain gap between the training set and the test environment may be encountered. In that case, we suggest to collect image data under specific application scenarios and use manual annotation to expand the generalizability of the images by fine-tuning to optimize the performance of the detection algorithm in order to expand the generalizability of Auto-LIA.

Moreover, the real-time computing technique is also one of the bottlenecks of Auto-LIA. In order to ensure the usability of the segmentation algorithm, our proposed gradient-based segmentation operation has a linear relationship with the number of pixels in the selected region, which leads to a longer computation time when the target region is large. We recommend that subsequent works strike a better balance between algorithmic effectiveness and algorithmic efficiency, and improve the usability of the Auto-LIA system by optimizing the segmentation algorithm.

## Conclusion

Existing LIA measurement techniques suffer from low automation, high destructiveness, and low refinement. In order to improve the efficiency of LIA collection without causing damage to the plant, we propose a full-auto method named Auto-LIA. Auto-LIA tackles a noninvasive RGB imaging method for data acquisition, which reduces the environmental disruption and cost of the process. We apply Auto-LIA to measure plants in different physiological states during the same time period, and the LIAs of the plants show different trends according to the attribute of plant water deficiency. Auto-LIA refines the management object from a plot to a single plant, which facilitates the development of precision agriculture management.

As for future applications, on the one hand, some previous work mentions that as a plant trait, LIA is an externalized expression of genotypic superiority or inferiority within the plant. We believe that Auto-LIA, a noninvasive collection, will provide multi-temporal dimensions of information for analyzing gene expression in plants. On the other hand, LIA is a feedback of leaf physical traits that can indirectly influence the efficiency of plant uptake of agricultural inputs and photosynthesis. We are optimistic that Auto-LIA will provide a means of continuously optimizing the plant growth process and increasing crop yields through sustainable regulation.

## Data Availability

We make our code and data publicly available at http://autolia.samlab.cn.

## Supplementary Materials

Supplementary 1 Supplementary Text References [51,52]
