# Supplementary material for: Auto-LIA: The Automated Vision-Based Leaf Inclination Angle Measurement System Improves Monitoring of Plant Physiology
Source: Plant Phenomics. 2024 Sep 11;6:0245. doi: 10.34133/plantphenomics.0245 (PMC11387749; doi:10.34133/plantphenomics.0245)
Supplement: Supplementary 1 — Supplementary Text References [51,52] [file plantphenomics.0245.f1.docx]

## Supplementary Materials

**Hardware Selection.** In order to reduce the labor input during data acquisition and to achieve non-invasive acquisition, we build a data acquisition system with two RGB cameras and an inclinometer as shown in Figure 2 (A). The data captured by the two cameras are used to reconstruct the spatial information of the plant. According to the principle of binocular imaging, the relative positions of the two cameras need to be consistent during the acquisition. In addition, the hardware information of the two RGB cameras has a large impact on the reconstruction effect [53], so the model, focal length, and other parameters of the two cameras should be kept the same. In this study, we select the Daheng MARS-1231-46G5C-P camera for image capture. With a resolution of 4K, the camera’s high image quality can support the acquisition of sufficiently fine-grained information, and the hardware provides the feasibility of integrating more applications. In addition, we chose lenses with a focal length of $30mm$, which have an optimal acquisition object distance of 3 to 5 meters. The imaging distance of 3 to 5 meters ensures a non-intrusive data acquisition process and facilitates expansion to UAV piggybacking. Besides visible cameras, an inclinometer is also applied in our data acquisition system, whose error reaches $\pm1^{\circ}$. This inclinometer is used to obtain the angle made by the acquisition system with the ground, and the conversion between the resulting angle and the LIA is described in Sec. 2.2.4.

**Camera Calibration.**

In order to achieve the transition from image space to real physical space, the data acquisition system also needs to obtain the internal and external parameters of the camera through calibration. Before the collection started, the algorithm proposed by Zhang [54] is used for calibration. With sub-pixel accuracy of feature point image coordinate extraction, this approach enables the relative error of camera calibration to be $1\%$ or less. Specifically, we synchronously capture 20 sets of chessboard images using two RGB cameras from different perspectives with relatively fixed bit positions, for finding the corner point information in each image. Second, the corner points are used for spatial estimation with algorithms implemented in OpenCV. The function ’calibrateCamera’ applies 20 images with the real size of corner points for measuring the internal parameters of cameras. Furthermore, the method ’stereoCalibrate’ is employed to metricize the rotational and translational associations between the two cameras in the binocular system. Finally, we obtain the effect of the sensor as well as the lens on the distortion of the image. After calibration, the relative position between the imaging devices will be used as a fixed parameter for correcting the distortion of the captured image and recovering the depth of the image. Particularly, to improve the efficiency of parameter computation while ensuring the accuracy of the camera estimation results, we further increase the prerequisites to be followed for camera deployment. We stipulate that the horizontal axis of the source plane is always kept parallel to the ground, which can reduce the spatial correlations that need to be dealt with during subsequent data processing.

**Plant Area Alignment.**

As shown in Figure 2 B-(a), the plant areas in 2 subgraphs are narrowed down to the visual area intended for stereo matching by the expansion and alignment operation, which effectively reduces the amount of data involved in the computation. The plant area alignment is mainly designed for the problem of inconsistency in the size of the plant areas provided in the 2 source images, which will cause the stereo matching task to be impossible. Specifically, we begin with finding the location of the center points of the 2 anchor boxes in order to reduce the excessive noise information introduced during the expansion of the plant areas. Then, we find the maximum height as well as the maximum width of the two associated regions and expand the anchors. For example, when the width and height of the two related areas are $(x_{1},y_{1})$ and $(x_{2},y_{2})$, where $x_{1}>x_{2}$ and $y_{1}<y_{2}$, the size of the expanded boxes will reach $(x_{1},y_{2})$ respectively. Eventually, we crop the source images of the target regions based on the coverage of the expanded boxes, and the cropped boxes of the same size will be involved in the subsequent depth estimation.

**Transform of Disparity Map.** The cropped images are fed into the RAFT model to obtain the disparity map between the pixel points of the two cropped images. However, the obtained disparity map here cannot be directly applied to spatial depth estimation. We combine the retained cropping frame information to transform the predicted disparity map in the plant region back to the real plane. Specifically, in the cropping frame, the point with the smallest horizontal coordinate is labeled as *x* in the corresponding image coordinate system, and the estimated disparity matrix *D* of each point, so that the corresponding coordinate of each pixel point in the target image is $D+x$. The image coordinate system in which the cropped images are located is recorded as *C*, and the coordinate of each coordinate in the cropped image in the source image is $C+x$. At last, the depth of each point can be converted by triangulation and spatial transformation, with the hardware parameters in the binocular system.

**Evaluation Platform.** For cost reduction and power consumption, we port all the algorithms to run on the CPU without using any GPU for acceleration. In this study, we deploy the LIA measurement system in PC: 32 Intel(R) Xeon(R) Gold 6230R CPU @ 2.10GHz.

**Model Adaptation.**

The whole process includes object detection, depth estimation, and image segmentation techniques in this study. As for object detection, we adopt the existing YoloV7 [42] algorithm for semantic information in images. In detail, we utilize the unaltered pre-trained model to detect the positions, object types, and confidence scores in the source image and crop the image based on its computational results to reduce the time-consuming.

After the detection by YoloV7 model with default parameters, we carry out post-processing for the detection results, which mainly includes category filtering and confidence filtering. The category filter fixes the class to which the detection frame belongs to the ’potted plant’ type to locate the plants. While filtering threshold 0.6 is used to provide quality plant localization by excluding lower quality plants. As far as depth estimation is concerned, we select the RAFT [43] algorithm to realize non-invasive spatial recovery. During the experiment, the disparity estimated by RAFT is combined with the external parameters of the binocular system for depth estimation. A large number of model default parameters are used in the implementation of 3D structural recovery with RAFT, e.g., the number of layers of the correlation pyramid is set to 4, the search window is set to 4, and so on. It should be noted that due to the difficulty of 3D real data acquisition, we chose the raft-kitti pre-trained model to accomplish the reconstruction of leaves in real scenes.
